# Mortality risk relationship using standard categorized BMI or knee-height based BMI – does the overweight/lower mortality paradox hold true?

**DOI:** 10.1007/s40520-024-02742-6

**Published:** 2024-04-08

**Authors:** Nivetha Natarajan Gavriilidou, Mats Pihlsgård, Sölve Elmståhl, Henrik Ekström

**Affiliations:** 1 Department of Dental Medicine, Division of Oral Diseases, Karolinska Institutet, Huddinge, Sweden; 2 Perinatal and Cardiovascular Epidemiology, Lund University Diabetes Centre, Department of Clinical Sciences in Malmö, Lund University, Malmö, Sweden; 3 Department of Clinical Sciences in Malmö, Division of Geriatric Medicine, Skåne University Hospital, Lund University, Malmö, Sweden; 4 Division of Geriatric Medicine, Skåne University Hospital, Lund University, Jan Waldenströms gata 35, Malmö, 205 02 Sweden

**Keywords:** Classic BMI, Knee-height based BMI, Mortality, Older adults, Population study

## Abstract

**Background:**

The body mass index (BMI) is prone to misclassification of obesity due to age-related height loss and resulting measurement errors. Knee-height based BMI (KH-BMI) has not been previously studied in relation to mortality risk in older adults.

**Aim:**

To evaluate the age- and sex-specific mortality risk relationship using classic BMI and knee height predicted BMI (KH-BMI) overweight and obesity in a 15-year follow-up study including older Swedish adults aged 60–93 years.

**Methods:**

A 15-year follow-up study among 2,786 individuals aged ≥ 60 years. Height, weight and KH were measured. KH-predicted height was estimated using formulated gender-specific equations. Classic BMI and KH-BMI (kg/m^2^) were calculated. Mortality data was obtained from the Swedish death registry. Questionnaires were used to collect data on obesity-related lifestyle factors and comorbidities.

**Results:**

Cox regression revealed that using the classic BMI, when comparing with the normal/underweight reference group, there was a mortality risk among overweight men (HR = 0.67, 0.52–0.87), overweight women (HR = 0.79, 0.65–0.97), and obese men (HR = 0.60, 0.41–0.89) aged ≥ 80 years old. Using the KH-BMI, only overweight men and overweight women aged ≥ 80 years had a lower mortality risk, men (HR = 0.71, 0.55–0.92); women (HR = 0.77, 0.62–0.95) after adjusting for obesity-related lifestyle factors and comorbidities.

**Discussion:**

There is evidence that obesity is overestimated by the BMI, in comparison with the KH-BMI classification. In terms of mortality risk and after adjusting for height, there remains a paradoxical protective association between overweight and mortality.

**Conclusion:**

Regardless of classic BMI or KH-BMI estimation, overweight men and women aged ≥ 80 years had a lower mortality risk compared to normal/underweight men and women ≥ 80 years.

## Introduction

Overweight and obesity are increasing worldwide among all age groups, posing a significant public health burden due to the associated elevated risk of cardiovascular diseases, type-2 diabetes, stroke, musculoskeletal disorders, cancers, functional impairment, and mortality [1, 2]. In older adults, aged 65 years and over, the relationship between overweight, obesity and morbidity does not seem as clear, since earlier studies have reported that overweight and a low degree of obesity (BMI 30 - <35) have a certain protective effect against co-morbidity [3, 4]. In addition, it has been suggested that neither overweight nor obesity increases the risk of all-cause mortality [5], particularly in the presence of comorbidities or acute medical problems [6].

Body mass index (BMI) is a widely employed tool for assessing nutritional status among all age groups. According to the World Health Organization (WHO), BMI within the range of 18.5–24.9 kg/m^2^ is considered healthy in relation to the morbidity and mortality risks associated with the higher and lower BMI categories [2, 7]. However, this conclusion was based on studies in young adults, including all ages > 20 years [2]. Gerontologists have claimed that this range is overly restrictive and recommend adjusting the cut-off values for older adults [8]. Such studies, however, require further validation [9]. In a study by Burman et al., it was reported that in older nursing home residents, a lower 2-year mortality was found among those who were overweight and obese compared to normal weight, while those who were obese had a lower mortality than those who were overweight [10]. Hence, irrespective of nutritional status, higher BMI was associated with lower mortality.

Another question is whether gender should be taken into account when deciding about BMI cut-off points. In a study by Carr et al. using BMI 21.0-24.9 as a reference, it was shown that among men, the lowest risk of all-cause mortality was found among those who were overweight and moderately obese (BMI 30-34.9), while in women only those who were overweight had a reduced mortality risk [11].

Aging is associated with loss of muscle mass, increased prevalence abdominal obesity, and fat redistribution [12, 13]. The BMI may therefore be a less reliable marker of adiposity among older adults, due to a lack of sensitivity in differentiating between fat and lean mass. Despite this, there is evidence that BMI is strongly associated with body fat levels as well as a significant predictor of chronic diseases [14]. However, as stated above, overweight or a lower degree of obesity among older adults may have a protective effect against morbidity [3, 4]. Nevertheless, the impact of BMI on mortality among older adults remains controversial. In addition, previous studies have described a wide variation in this relationship, from a direct positive, a U or J-shaped, to an inverse association [15–17]. Hence, there is a need for further examination of this association in larger, generalizable population samples.

In addition, as BMI is often used in clinical work to assess health status, it is important that the evaluation is made as accurately as possible. It may apply to the perioperative assessment of health status, of which BMI forms an important part [18], to nutritional status when designing weight loss programs for determining the frailty of older adults in rehabilitation programs or to the risk of common diseases such as diabetes, arthritis or high blood pressure [12].

To our knowledge, there are no previous studies that have adequately addressed the consequences of height loss and height estimation errors among older adults. Besides the above-mentioned consequences of aging on body composition, there are also phenotypic modifications, such as height loss and kyphosis, where women tend to lose more height than men, partly due to a greater risk of osteoporosis, that can cause problems in height measurements [19–21]. This could in turn lead to misclassification and false estimations of the prevalence of underweight and obesity in older adults. Therefore, the use of established surrogate measures such as knee-height (KH) and demispan (the distance from the middle of the sternal notch to the tip of the middle finger) has been suggested [19, 22–24]. In contrast to total body height, the length of the tibia (or other long bones) is not influenced by aging. Knee-height can be used as a proxy to calculate maximum adult height.

Previous studies have shown that classic BMI height estimates, obtained by the standard method of height measurement using a measuring tape and erect posture, could overestimate obesity prevalence twice as much compared to KH or demispan [19]. However, among those aged 80 years and older, there are no previous studies on these differences in BMI classification or what they mean for the association between overweight/obesity and mortality [9].

Thus, there is a need for age-stratified survival studies using classic BMI versus proxy BMI measurements predicted using KH.

## Aims

To evaluate the age and sex-specific mortality risk relationship using classic BMI and knee-height predicted BMI (KH-BMI) overweight and obesity in a 15-year follow-up study of older Swedish adults aged 60–93 years.

## Methods

### Study population

This is a 15-year follow-up study within a longitudinal, general population-based survey called Good Aging in Skåne (GÅS), part of the Swedish National Study on Aging and Care (SNAC) [25, 26]. The GÅS study is conducted among a heterogeneous sample of men and women from five urban and rural municipalities in Region Skåne. The National Population Registry was used to randomly invite the participants by letter. Inability to speak and understand Swedish, bedridden patients and those using a wheelchair were excluded. Predefined target populations were invited for the age cohorts 60, 66, 72, 78, 81, 84, 87, 90, and 93 years, with an oversampling of the youngest and the oldest cohorts. Randomization was performed with an excess intake of the youngest (60 and 66 years) and the oldest (87, 90, and 93 years) age groups. The purpose of a larger randomization among the 60-year-olds was to create a larger base for longitudinal studies, while that of a randomized over-intake among the 87, 90, and 93 years old was to obtain a larger statistically motivated base. The GÅS baseline examination (2001–2004) consisted of 2,931 participants, aged 60–99 years. The response rate was 60%. Of these participants, 2,786 persons (1,543 women, 1,243 men) had valid KH and BMI measurements and constituted the subjects in the present study. They were followed up for 15 years. During this period, 1,296 participants died and 54 emigrated from the country.

### Data collection

All participants were examined at a research center, with the exception of frail older individuals, to whom home visits were offered. Informed consent was provided by all participants. The survey, medical examination, and physical functioning tests were conducted by qualified physicians and nurses. The close-ended questionnaire was used to investigate socio-demographics, physical health, mental health, and social factors.

### Descriptive variables

Data on age, sex, residence, marital status, education, smoking habits, and physical activity was obtained from the survey. The population was categorized into three age groups: 60–69 years, 70–79 years, and ≥ 80 years. Other categories included:

#### Marital status

single, married, divorced, or living with a partner; Residence: urban or rural;

#### Education

primary, lower, secondary, or higher education; Smoking: smoker (regular and irregular), non-smoker, and ex-smoker.

#### Physical activity (intensity/grades of exertion)

mostly sedentary (none/very light activity), moderate (1–2 h/week), mild (around 2–4 h/week) and heavy (3 h/week or more).

### Underlying medical conditions

Information on the diagnosed medical conditions under study was obtained from medical records, medical examination, and functional tests. The diseases and conditions included were those particularly common in older adults: myocardial infarction, stroke, diabetes, tuberculosis, asthma, chronic obstructive pulmonary disease, osteoporosis, hip fracture, arthritis, dementia, Parkinson’s disease, depression, and cancer.

### Mortality

Information about survival status and the date of death for the deceased population was obtained from the Swedish Civil Registry. The dataset used was updated until 2016. Of the 2,786 participants in our study, 1,296, mean age 80.5 (± 9.1) years, died 6.3 (± 3.8) years after the examination. Among those who died during the follow-up, 17.1% were aged 60–69 years, 21.5% 70–79 years, and 61.4% ≥80 years (Table 1).

**Table 1:** Number of men and women in different age groups at baseline examination and number of deaths at 15-year follow-up

Participants	Baseline	Deceased at 15-years follow-up
Study sample (years)		
60–69, n (%)	1356 (48.7)	222 (16.4)
70–79, n (%)	543 (19.5)	278 (51.2)
≥ 80, n (%)	887 (31.8)	796 (89.7)
Total	2786 (100)	1296 (46.5)
Men (years)		
60–60, n (%)	676 (54.4)	134 (19.8)
70–79, n (%)	244 (19.6)	148 (60.7)
≥ 80, n (%)	323 (26.0)	304 (94.4)
Total	1243 (100)	586 (41.7)
Women (years)		
60–69, n (%)	680 (44.1)	88 (12.9)
70–79, n (%)	299 (19.4)	130 (43.5)
≥ 80, n (%)	564 (36.6)	492 (87.2)
Total	1543 (100)	710 (46.0)

### Anthropometric measurements

Height, weight, and KH were measured based on validated protocols [27–29]. Height was measured using a measuring tape with the individual standing erect with shoulder blades, buttocks, and heels against a wall and a straight fixed gaze. Arms were by the sides, feet flat, and heels together. Measurements were made in cm with one decimal digit.

Weight (in kg) was measured with a precision scale in the morning with light clothes and no shoes after voiding bowels and bladder.

KH (in cm) was measured using a caliper consisting of a vertical scale with two horizontal blades at each end. The subject was in a recumbent position, with neck and back relaxed, left leg lifted and knee bent at 90°. One of the caliper blades was positioned under the heel of the left foot and the other on the anterior surface of the left thigh just above the condyles of the femur and proximal to the patella. The shaft of the caliper was held parallel to that of the tibia. The measurement was repeated twice, and the average noted. If seated, the leg was supported so that the knee and ankle were at a 90° angle.

Measurements were made on the left side, except in cases of amputation, paralysis, or contracture.

### Classic BMI and KH-BMI

BMI was measured using the formula: weight in kg divided by the square of height in meters. Unit: kg/m^2^. In our previous published article addressing BMI misclassifications due to inaccurate height estimation among older adults, we presented the gender-specific equations to predict body height based on KH values. The KH based prediction equation was used to calculate KH-BMI [19]. The equations were:

$$\eqalign{& Height\, = \,115.23\, + \,1.16\, \times \,KH\,\left( {Men} \right); \cr & Height\, = \,104.52\, + \,1.23\, \times \,KH\,\left( {Women} \right) \cr}$$

Based on the WHO guidelines of categorizing BMI levels, we divided our study population into four categories: normal: 18.5 ≤ BMI < 25 kg/m^2^, underweight: BMI < 18.5 kg/m^2^, overweight: 25 ≤ BMI < 30 kg/m^2^, and obese: BMI ≥ 30 kg/m^2^. With only 32 underweight participants (1%), this category was merged with the ‘normal BMI’ category. The tests performed in which this merged category served as a reference were repeated using only the normal BMI group and excluding the underweight group. To check that the merger would not affect the overall result, we compared the survival rates between the two groups. In the group with underweight and normal weight as a reference, the Hazard Ratio (HR) for overweight and obese was (HR = 0.63, CI 95% 0.56–0.71) and (HR = 0.55, CI 95% 0.47–0.75) respectively, and in the group with only normal weight as a reference, the HR for overweight and obese was (HR = 0.67, CI 95% 0.59–0.75) and (HR = 0.59, CI 95% 0.50–0.70), respectively.

### Statistical analyses

Socio-demographic characteristics of the study population separately classified into normal/underweight, overweight, and obese groups by classic BMI and KH-BMI are presented.

Age and gender-specific Cox regression models were formulated. The model was adjusted for the following variables: marital status, residence, education, smoking, physical activity, and number of diagnosed medical conditions. The respective reference groups for the variables included in the regression analysis were normal/underweight BMI, age 60–69 years, married, urban residence, higher than secondary education level, non-smoker, and heavy physical activity. An attrition analysis was conducted to examine and compare non-participants and participants regarding age, and any difference was tested with Student’s T-test. Critical p-values were ≤ 0.01 and ≤ 0.05. The data analyses were performed by the first author (N.N.G) and statistician (M.P.).

## Results

Our study included 2,786 subjects, 44.4% men and 55.6% women, mean age 71.84 ± 10.4 years. Of these, 57.8% were married, 17.1% smokers, 22.2% mostly sedentary and 25.5% moderately active, while 54.3% had at least a primary education. The average number of diagnosed medical conditions was 1.00 (± 0.99). A description of the population after stratification into BMI categories is presented in Tables 2 (classic BMI classification) and 3 (KH-BMI classification).

**Table 2:** Description of the study population stratified by classic BMI classification including Swedish older adult men and women participants from the baseline examination (year 2001) of Good Aging in Skåne (GÅS) study followed up until 2016

	Men			Women			
	Normal	Overweight	Obese	Normal	Overweight	Obese	
Number of subjects	376	621	246	620	596	327	
Age at examination	74.0 (11.2)	70.5 (9.4)	68.7 (8.9)	74.7 (11.5)	73.7 (10.4)	72.4 (9.9)	
Age groups							
60–69 years	171(45.5)	347 (55.9)	158 (64.2)	268 (43.2)	257 (43.1)	155 (47.4)	
70–79 years	62 (16.5)	137 (22.1)	45 (18.3)	97 (15.6)	125 (21.0)	77 (23.5)	
80 + years	143 (38.0)	137 (22.1)	43 (17.5)	255 (41.1)	214 (35.9)	95 (29.1)	
Residence							
Rural	130 (34.6)	252 (40.6)	87 (35.4)	194 (31.3)	199 (33.4)	140 (42.8)	
Urban	246 (65.4)	369 (59.4)	159 (64.6)	426 (68.7)	397 (66.6)	187 (57.2)	
Marital status							
Single	117 (32.1)	128 (20.9)	72 (29.8)	348 (57.8)	301 (52.1)	179 (56.6)	
Married	248 (67.9)	483(79.1)	170 (70.2)	254 (42.2)	277 (47.9)	137 (43.4)	
Education							
Primary or lesser	170 (46.8)	298 (49.0)	133 (55.4)	333 (55.7)	336 (58.3)	195 (62.1)	
Secondary	104 (28.7)	186 (30.6)	67 (27.9)	157 (26.3)	157 (27.3)	80 (25.5)	
University or higher	89 (24.5)	124 (20.4)	40 (16.7)	108 (18.1)	83 (14.4)	39 (12.4)	
Smoking							
Smokers	84 (23.2)	98 (16.1)	39 (16.2)	126 (21.0)	77 (13.4)	40 (12.7)	
Ex-smoker	166 (45.9)	309 (50.7)	144 (59.8)	150 (25.0)	156 (27.1)	99 (31.4)	
Non-smoker	112 (30.9)	202 (33.2)	58 (24.1)	324 (54.0)	342 (59.5	176 (55.9)	
Physical activity intensity							
Mostly sedentary	90 (25.0)	124 (20.5)	68 (28.2)	125 (21.0)	111 (19.4)	78 (24.8)	
Mild (2–4 h/week)	164 (45.6)	273 (45.1)	103 (42.7)	277 (46.6)	297 (51.9)	154 (49.0)	
Moderate (1–2 h/week)	85 (23.6)	146 (24.1)	52 (21.6)	176 (29.6)	152 (26.6)	74 (23.6)	
Heavy (3 h/ week or more)	21 (5.8)	62 (10.2)	18 (7.5)	17 (2.9)	12 (2.1)	8 (2.5)	
Diagnoses^a^	0.8 (1.0)	0.7 (0.82)	0.71 (0.9)	1.00 (1.1)	0.93 (1.0)	1.0 (1.1)	
Number of deaths during follow up	217 (57.7)	270 (43.5)	99 (40.2)	301 (48.5)	260 (43.6)	149 (45.6)	

^a^Number of diagnosed medical conditions (myocardial infarction, stroke, diabetes, tuberculosis, asthma, chronic obstructive pulmonary disease, osteoporosis, hip fracture, arthritis, dementia, Parkinson’s disease, depression and cancer)

**Table 3:** Description of the study population stratified by knee height predicted BMI classification including Swedish older adult men and women participants from the baseline examination (year 2001) of Good Aging in Skåne (GÅS) study followed up until 2016

	Men			Women			
	Normal	Overweight	Obese	Normal	Overweight	Obese	
Number of subjects	470	551	222	774	526	243	
Age at examination	74.5 (10.9)	70.2 (9.2)	67.2 (8.3)	75.9 (11.2)	72.5 (10.1)	69.8 (8.8)	
Age groups							
60–69 years	199 (42.3)	319 (57.9)	158 (71.2)	286 (37.0)	250 (47.5)	144 (59.3)	
70–79 years	91 (19.4)	114 (20.7)	39 (17.6)	129 (16.7)	114 (21.7)	56 (23.0)	
80 + years	180 (38.3)	118 (21.4)	25 (11.3)	359 (46.4)	162 (30.8)	43 (17.7)	
Residence							
Rural	198 (42.1)	211 (38.3)	60 (27.0)	266 (34.4)	183 (34.8)	84 (34.6)	
Urban	272 (57.9)	340 (61.7)	162 (73.0)	508 (65.6)	343 (65.2)	159 (65.4)	
Marital status							
Single	138 (30.3)	121 (22.2)	58 (26.5)	446 (59.9)	263 (50.9)	119 (50.6)	
Married	317 (69.7)	423 (77.8)	161 (73.5)	298 (40.1)	254 (49.1)	116 (49.4)	
Education							
Primary or lesser	229 (50.6)	263 (48.6)	109 (50.2)	434 (58.7)	289 (56.0)	141 (60.5)	
Secondary	129 (28.5)	168 (31.1)	60 (27.6)	187 (25.3)	146 (28.3)	61 (26.2)	
University or higher	95 (21.0)	110 (20.3)	48 (22.1)	118 (16.0)	81 (15.7)	31 (13.3)	
Smoking							
Smokers	94 (20.8)	93 (17.1)	34 (15.6)	138 (18.6)	70 (13.6)	35 (14.9)	
Ex-smoker	217 (48.1)	268 (49.4)	134 (61.5)	173 (23.4)	146 (28.3)	86 (36.6)	
Non-smoker	140 (31.0)	182 (33.5)	50 (22.9)	429 (58.0)	299 (58.1)	114 (48.5)	
Physical activity intensity							
Mostly sedentary	107 (23.8)	121 (22.4)	54 (24.9)	166 (22.6)	96 (18.7)	52 (22.1)	
Mild (2–4 h/week)	206 (45.9)	238 (44.1)	96 (44.2)	359 (49.0)	253 (49.3)	116 (49.4)	
Moderate (1–2 h/week)	104 (23.2)	127 (23.5)	52 (24.0)	191 (26.1)	150 (29.2)	61 (26.0)	
Heavy (3 h/ week or more)	32 (7.1)	54 (10.0)	15 (6.9)	17 (2.3)	14 (2.7)	6 (2.6)	
Diagnoses^a^	0.84 (1.0)	0.66 (0.8)	0.65 (0.8)	1.02 (1.1)	0.91 (1.0)	0.99 (1.0)	
Number of deaths during follow up	271 (57.7)	238 (43.2)	77 (34.7)	413 (53.4)	204 (38.8)	93 (38.3)	

^a^Number of diagnosed medical conditions (myocardial infarction, stroke, diabetes, tuberculosis, asthma, chronic obstructive pulmonary disease, osteoporosis, hip fracture, arthritis, dementia, Parkinson’s disease, depression and cancer)

**Fig. 1:**
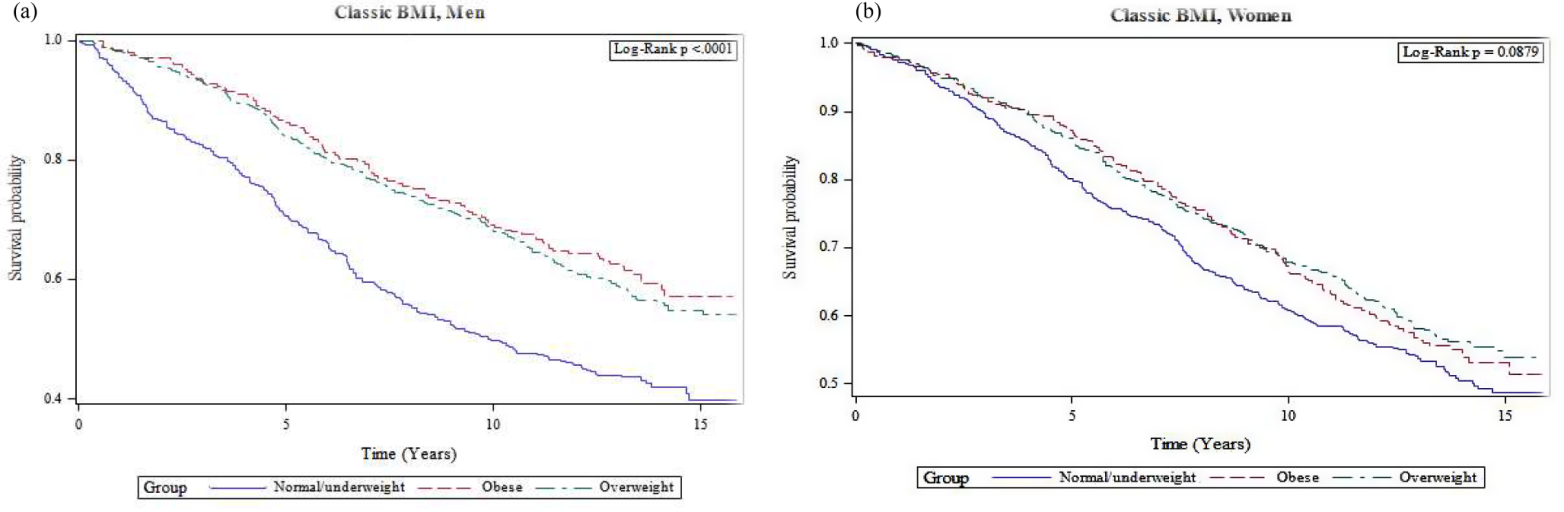
(**a**) Cox proportional hazards model for overall survival duration among normal-, overweight and obese men according to classic BMI. (**b**) Cox proportional hazards model for overall survival duration among normal-, overweight and obese women according to classic BMI

**Fig. 2:**
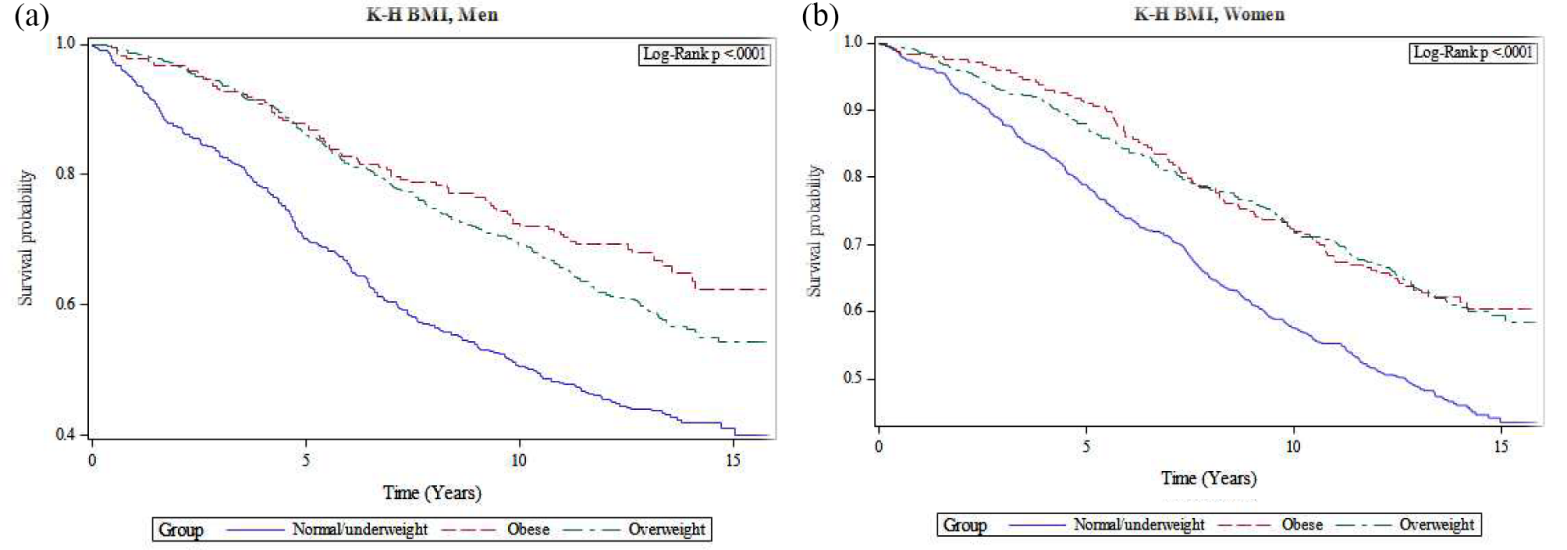
(**a**) Cox proportional hazards model for overall survival duration among normal-, overweight and obese men according to K-H BMI. (**b**) Cox proportional hazards model for overall survival duration among normal-, overweight and obese women according to K-H BMI

Irrespective of the classic BMI or KH-BMI method, being overweight and obese led to a lower mortality risk in both men and women in comparison with the reference group (Figs. 1a and b and 2a and b). After adjusting for confounders, there was still a lower mortality risk among overweight men (BMI: HR = 0.72, 0.60–0.87); (KH-BMI: HR = 0.81, 0.67–0.97) and women (BMI: HR = 0.87, 0.70–0.98); (KH-BMI: HR = 0.81, 0.67–0.97). According to classic BMI, there was also a lower mortality risk among obese men (HR = 0.71, 0.56–0.93) (Table 4).

**Table 4:** Hazard ratios for all-cause mortality of overweight and obesity according to knee-height predicted BMI,- and classic BMI levels among men and women in the GÅS study population

	Men			Women		
	Cases of death n (%)	Crude HR.	Adjusted HR^a^	Cases of death n (%)	Crude HR	Adjusted HR^a^
Classic BMI						
Normal/ underweight	217 (37.0)	Ref	Ref	301 (42.4)	Ref	Ref
Overweight	270 (46.1)	0.63 (0.52–0.76)	0.72 (0.60–0.87)	260 (36.6)	0.86 (0.72–1.02)	0.87 (0.70–0.98)
Obese	99 (16.9)	0.56 (0.43–0.71)	0.71 (0.56–0.93)	149 (21.0)	0.91 (0.74–1.11)	1.03 (0.84–1.27)
K-H BMI						
Normal/ underweight	271 (46.2)	Ref	Ref	413 (58.2)	Ref	Ref
Overweight	238 (40.6)	0.63 (0.52–0.75)	0.81 (0.67–0.97)*	204 (28.7)	0.65 (0.53–0.75)	0.81 (0.67–0.97)*
Obese	77 (13.1)	0.47 (0.37–0.61)	0.81 (0.61–1.06)	93 (13.1)	0.62 (0.49–0.79)	1.07 (0.84–1.37)

^a^Adjusted for marital status, residence, education, smoking, physical activity, number of diagnosed medical conditions (myocardial infarction, stroke, diabetes, tuberculosis, asthma, chronic obstructive pulmonary disease, osteoporosis, hip fracture, arthritis, dementia, Parkinson’s disease, depression and cancer), *p* ≤ 0.01, *p ≤ 0.05

Table 5 presents the mortality risks derived from classic BMI and KH-BMI, respectively, after age stratification and adjusting for confounders. According to classic BMI, there was a lower mortality risk in overweight men (HR = 0.67, 0.52–0.87); overweight women (HR = 0.79, 0.64–0.97), and obese men (HR = 0.60, 0.41–0.89) aged ≥ 80 years. According to KH-BMI, compared to the reference group, a lower mortality risk was observed in overweight men and women (men: HR = 0.71, 0.55–0.92; women: HR = 0.77, 0.62–0.95) aged ≥ 80 years. Irrespective of the method of BMI estimation, obese men and women aged ≥ 80 years did not have an increased mortality risk. Furthermore, the survival benefit was not statistically significant when using KH-BMI.

The attrition analysis showed that the mean age of participants (72.9, SD = 10.6) and non-participants (76.0, SD = 11.6) differed significantly.

**Table 5:** Hazard ratios (HR) for all-cause mortality according to knee-height predicted BMI, - and classic BMI levels for different age groups among men and women in the GÅS study population

			Men			Women	
Classic BMI	Age decade	BMI mean (sd)	Crude HR	Adjusted HR^a^	BMI mean (sd)	Crude HR.	Adjusted HR^a^
Overweight	60–69 years	27.26 (1.41)	0.81 (0.54–1.20)	0.84 (0.56–1.26)	27.18 (1.44)	1.19 (0.71-2.00)	1.04 (0.61–1.77)
Obese		33.30 (2.98)	0.77 (0.47–1.25)	0.71 (0.43–1.17)	33.65 (3.42)	1.72 (1.01–2.95)	1.14 (0.64–2.04)
Overweight	70–79 years	27.16 (1.40)	0.68 (0.46–0.99)	0.71 (048-1.06)	27.28 (1.38)	0.88 (0.58–1.34)	0.94 (0.61–1.46)
Obese		32.92 (4.11)	0.83 (0.51–1.36)	0.89 (0.53–1.48)	33.61 (3.46)	1.36 (0.87–2.12)	1.23 (0.78–1.95)
Overweight	80 + years	27.06 (1.34)	0.69 (0.53–0.88)	0.67 (0.52–0.87)	27.17 (1.49)	0.75 (0.61–0.92)	0.79 (0.64–0.97)*
Obese		32.85 (5.59)	0.66 (0.46–0.96)*	0.60 (0.41–0.89)	32.63 (2.90)	0.78 (0.60–1.02)	0.83 (0.63–1.09)
K-H BMI							
Overweight	60–69 years	27.31 (1.41)	0.87 (0.58–1.29)	0.91 (0.61–1.37)	27.30 (1.43)	0.91 (0.55–1.54)	0.90 (0.54–1.50)
Obese		33.43 (3.39)	0.92 (0.58–1.48)	0.94 (0.58–1.53)	33.46 (3.10)	1.41 (0.83–2.42)	0.97 (0.54–1.73)
Overweight	70–79 years	27.10 (1.38)	0.76 (0.53–1.08)	0.84 (0.57–1.22)	27.22 (1.40)	0.89 (0.60–1.33)	0.88 (0.58–1.33)
Obese		33.14 (4.18)	0.70 (0.42–1.18)	0.71 (0.41–1.22)	33.21 (3.41)	1.38 (0.87–2.18)	1.31 (0.81–2.13)
Overweight	80 + years	27.01 (1.41)	0.77 (0.60–0.98)*	0.71 (0.55–0.92)	27.19 (1.53)	0.76 (0.62–0.94)	0.77(0.62–0.95)
Obese		32.52 (5.06)	0.79 (0.51–1.24)	0.73 (0.46–1.15)	32.41 (2.51)	0.85 (0.60–1.21)	0.87 (0.61–1.25)

^a^Adjusted for marital status, residence, education, smoking, physical activity, number of diagnosed medical conditions (myocardial infarction, stroke, diabetes, tuberculosis, asthma, chronic obstructive pulmonary disease, osteoporosis, hip fracture, arthritis, dementia, Parkinson’s disease, depression and cancer), *p* ≤ 0.01, *p ≤ 0.05

## Discussion

In this population study, we followed 2,786 participants for 15 years and investigated the association between BMI and mortality risk in older adults aged ≥ 60 years. We aimed to address the problem of BMI misclassification due to age-related height loss and inaccurate height estimates among older adults using KH-BMI as a proxy measure.

Our results suggest that regardless of the BMI estimation method, overweight older men and women have a lower mortality risk, compared to normal/underweight individuals after adjusting for confounders, such as smoking, education, physical activity, residence, marital status, and comorbidities [6]. Irrespective of the method, obesity in men and women aged ≥ 80 years did not lead to an increased mortality risk. However, this survival benefit for obese men > 80 years was only observed using classic BMI and not KH-BMI. The relationship was not significant when classified using KH-BMI. This could be due to BMI misclassification of those individuals who were falsely classified as obese by classic BMI and later reclassified as overweight by KH-BMI. Nevertheless, further investigation is recommended to rule out the potential problem of lower statistical power in the obese group. However, in our study, the mean classic BMI was within grade 1 level of obesity (men: 32.6 kg/m^2^, women: 32.9 kg/m^2^), most likely supporting the misclassification explanation. Although the prevalence of obesity decreases with age, lack of higher obesity grades could possibly be due to attrition of morbidly obese subjects, which could also raise concern regarding the representation of obesity in the study [30, 31].

Our findings are consistent with some previous studies using classic BMI, which have shown a lower mortality risk in overweight and mildly obese (BMI 30–35) older adults as well as an increased mortality risk at BMI > 35 [14, 32–36]. A meta-analysis by Flegal et al., which used the WHO cut-off points for overweight and obesity, reported hazard ratios (95% CI) of 0.90 (0.86–0.95), 0.88 (0.69–1.12), and 1.28 (0.93–1.76) for a BMI of 25 – <30, 30 - < 35, and ≥ 35, respectively, suggesting a protective effect of overweight and modest obesity in older adults [32].

Aune et al.’s meta-analysis of 230 cohort studies with > 3.74 million deaths among more than 30.3 million participants indicates that lower mortality risk in overweight people is likely to be confounded by smoking and pre-diagnostic weight loss [17]. However, the study was not confined to older adults. A study by Bowman et al. including participants aged over 65 years showed that after adjusting for confounders, i.e., smoking, alcohol consumption, and socioeconomic status, obesity was associated with increased mortality, up to the age of 84 years [37].

Another meta-analysis including older adults aged > 65 years showed no increase in mortality risk among those who were overweight in comparison with normal BMI individuals (estimated risk 1.00 with 95% confidence interval: 0.97–1.03) as well as a moderate risk increase for those who were obese (1.10, 1.06–1.13) [9]. No studies have been previously conducted to investigate the effect of BMI misclassification due to height loss in older adults.

There is clear evidence for the overestimation of obesity by classic BMI, in comparison with the KH-BMI classification. In terms of mortality risk, we can state that after adjusting for height issues among older adults, there remains a paradoxical protective association between overweight and mortality but that the effect of moderate and severe obesity should be investigated further using KH-BMI. The lower statistical power in the obese group is apparently due to the fact that some individuals who were incorrectly classified as obese by classic BMI were overweight according to KH-BMI. Our KH-BMI findings confirm the J-shaped association between BMI and mortality, with a clear survival benefit in the overweight older adult population [19, 38]. After accounting for the effect of height changes measured with KH-BMI, it is important to understand the biological compartments that contribute to body weight, namely the fat mass (adiposity) and the fat-free mass (muscle mass). The protective effect of overweight might be attributed to resilience among individuals who survived the adverse effects of elevated BMI in middle age. This resilience could be strengthened by good healthcare, the protective metabolic effects of increased lean body mass, availability of nutritional reserves to support aging as well as age-related conditions [9, 12, 39]. A higher BMI among older adults, particularly the oldest old, is likely to indicate sufficient lean mass rather than fat stores (adiposity) [40]. Sometimes, higher adiposity is prevalent with stable BMI due to age-associated muscle loss [41]. This condition is known as sarcopenic obesity, which, like sarcopenia in the absence of obesity, can cause loss of muscle strength and function [42, 43]. Lean mass is independently associated with lower mortality and considered an indicator of a healthy lifestyle during younger years that included muscle-building levels of physical activity [9, 44]. Better maintained muscle mass can also reduce the risk of metabolic syndrome as well as cardiovascular diseases, elevated blood lipids, insulin resistance, hypertension, and obesity [45]. In addition, since skeletal muscle is the largest metabolic tissue in the body and has a crucial role in the disposal of glucose, a better maintained muscle mass can also reduce the risk of the metabolic syndrome as well as cardiovascular diseases, elevated blood lipids, insulin resistance, high blood pressure and obesity [46].

## Limitations

Our study is, however, not free from weaknesses. Firstly, the reliability of using knee height to determine body height is extremely important for the study. Provided that measurement is carried out correctly – which we have no reason to doubt – we think estimated height is a more accurate method even though previous studies have argued that the difference between estimated and measured height in older adults was non statistically significant [47, 48]. Secondly, potential limitations of using BMI as a marker of obesity call for attention, particularly the confounding effect of abdominal obesity that is positively associated with mortality [49, 50]. It could therefore be important to study the role of waist circumference in future research. However, this was beyond the scope of the present study. Classic BMI is still a widely accepted measure of body fat, an excellent marker of overall obesity, and a predictor of specific and all-cause mortality [50]. Thirdly, based on the known association between underweight and mortality in older adults, it could be possible that the relative risk reduction in the overweight/obese group could be explained by the elevated mortality in the reference group (that also included the underweight subjects [51]. Nevertheless, because there were only 32 underweight participants in our study, of whom nine died during follow-up (the total number of deaths in the reference group was 518), it could imply only a minimal risk reduction effect. Prior to the analysis stage were aware of the potential bias the lack of underweight participants could cause during the analysis stage and, as mentioned earlier, tested it by restricting the reference group to subjects with a normal BMI. We found no significant change in the results, confirming that the observed effect was not affected by any selection bias in the reference group. Fourthly, the data on the cases of death was obtained from the National Death Registry and does not include the cause of death. It could be more relevant to use updated data on cause of death based on autopsy reports. However, this might not have affected our study results, as the present study investigated all-cause mortality. Fifthly, it can be seen as a shortcoming that we did not consider other cut-offs for BMI. A study by Di Renzo et al. showed that, in a clinical setting, a BMI cut-off point 27.27 kg/m2 predicted obesity in middle-aged and older adults [52]. However, as the issue is still being debated, we chose to use the cut-offs recommended by the WHO. Finally, the lack of significant results among 60 and 70-year-olds might be due to lack of a large number of deaths and therefore statistical power.

Another limitation is that we did not consider that possible cohort effects may have influenced the results. 60 and 90-year-olds have grown up during different periods with different conditions for survival, for example access to healthcare. One can speculate that it is not the excess weight that is protective in older adults, but that they belong to the cohort that can cope with being overweight.

The attrition rate amounted to 40% and the mean age of non-participants and participants was 72.9 years, SD = 10.6, and 76.0 years, SD = 11.6, respectively. We cannot rule out that those who declined participation were the frailest with an overrepresentation of underweight or normal weight, and probably with a shorter expected survival time. This might suggest that if the non-participants had been included, our results on longer survival among the overweight would have been strengthened.

## Strengths

The main strength of this study is being the first to account for the effect of BMI misclassification on body mass-mortality risk associations with the help of a useful proxy measure, the KH-BMI. The effectiveness of KH in the prediction of height cannot be overlooked [19, 22], particularly in terms of the ease of measurement and minimal need for patient cooperation in clinical settings.

Other noteworthy strengths of this study that increase the validity of the results are the length of follow-up being ≥ 10 years, age and gender stratification, and an objective method of measurement of height and weight (self-reported measures being prone to social desirability bias), as well as adjusting for underlying medical conditions considered as competing risk factors for longevity [17].

## Conclusion

Based on both classic BMI and K-H BMI, we found a lower mortality risk in overweight men and women ≥ 80 years compared to normal weight. When comparing the two methods within the same age group, K-H BMI showed a slightly higher all-cause mortality risk among men, compared to classic BMI. The opposite was found among women; K-H BMI showed a slightly lower all-cause mortality risk compared to classic BMI. Furthermore, no increased all-cause mortality risk was noted among obese ≥ 80-year-old men and women based on either classic BMI or K-H BMI. Hence, when planning weight modification programs and health promotion strategies for older adults, it is important to address this overweight paradox along with other risky lifestyle factors and comorbidities.

## Data Availability

The authors confirm that the data supporting the findings of this study is available within the article.
